# Optically Stimulated Luminescent Response of the LiMgPO_4_ Silicone Foils to Protons and Its Dependence on Proton Energy

**DOI:** 10.3390/ma16051978

**Published:** 2023-02-28

**Authors:** Michał Sądel, Leszek Grzanka, Jan Swakoń, Jakub Baran, Jan Gajewski, Paweł Bilski

**Affiliations:** 1 Institute of Nuclear Physics, Polish Academy of Sciences, 31-342 Kraków, Poland; 2 Faculty of Physics, Astronomy and Applied Computer Science, Jagiellonian University, 30-348 Kraków, Poland

**Keywords:** luminescent materials, optically stimulated luminescence (OSL), relative luminescence efficiency, LiMgPO_4_, proton radiotherapy

## Abstract

Modern radiotherapy (RT) techniques, such as proton therapy, require more and more sophisticated dosimetry methods and materials. One of the newly developed technologies is based on flexible sheets made of a polymer, with the embedded optically stimulated luminescence (OSL) material in the form of powder (LiMgPO_4_, LMP) and a self-developed optical imaging setup. The detector properties were evaluated to study its potential application in the proton treatment plan verification for eyeball cancer. The data showed a well-known effect of lower luminescent efficiency of the LMP material response to proton energy. The efficiency parameter depends on a given material and radiation quality parameters. Therefore, the detailed knowledge of material efficiency is crucial in establishing a calibration method for detectors exposed to mixed radiation fields. Thus, in the present study, the prototype of the LMP-based silicone foil material was tested with monoenergetic uniform proton beams of various initial kinetic energies constituting the so-called spread-out Bragg peak (SOBP). The irradiation geometry was also modelled using the Monte Carlo particle transport codes. Several beam quality parameters, including dose and the kinetic energy spectrum, were scored. Finally, the obtained results were used to correct the relative luminescence efficiency response of the LMP foils for monoenergetic and spread-out proton beams.

## 1. Introduction

With the recent developments in modern radiotherapy (RT) techniques, such as proton therapy (PRT), where the range of the particle track is critical, and particles stop in a well-defined treated volume of the patient’s body, the demand for patient-specific dosimetric verification systems, e.g., for quality assurance (QA), is increasing. Since state-of-the-art PRT delivers a high-resolution spatially designed three-dimensional (3D) dose distribution, it should also be verified in 3D. Thus, more and more sophisticated dosimetry methods are required to validate complex treatment plans properly. These also imply the development of new kinds of dosimetry techniques and materials. The ionisation chambers are standard tools for QA systems in RT techniques such as proton radiotherapy. Other very popular tools are passive luminescent detectors, such as optically stimulated luminescent (OSL) discs, widely applied in personal dosimetry, phantom measurements, or in vivo dosimetry [1,2]. The principles of the OSL phenomena rely on the ability to register the ionising radiation through a specific long-lived trapped state populated during irradiation. This population can be read out in a separate process by subjecting the OSL material to a stimulating light field, which triggers the luminescence, providing a time-dependent signal (so-called OSL decay curve) proportional to the dose. The process is typically recorded by a photomultiplier tube (PMT) inside an OSL reader, which is equipped with light sources (diode/laser) of a wavelength that is specially matched to the OSL material used. One may state that the dose information provided by this technique always gives point-based information (one-dimensional). Concerning the 2D or 3D dosimetry systems applied for RT applications, different OSL materials have been evaluated for dose mapping in the past two decades [3]. An example list of the most promising materials can be listed: SrS [4], BeO [5], KCL [6], LiF:Mg,Cu,P [7], Al_2_O_3_:C [8], and LiMgPO_4_ [9,10,11,12].

Recently developed new dosimetry technology is based on the LiMgPO_4_ (LMP) doped with Tb (1.2 mol%) and B (10 mol%) [12]. For the first time, the LMP material was synthesised in powder form by a solid-state reaction in the air by Dhabekar and co-workers [13] in 2011. The luminescence emission of LMP is related to the d-f transitions of Tb^3+^ ions [13,14,15,16,17,18,19,20]. Its promising dosimetric properties, high radio-sensitivity, broad linear dose–response (up to 1 kGy), good repeatability, and acceptable fading allow for it to be considered as the potential alternative for the commercially available OSL materials.

In the prototype technology, the LMP powder is embedded homogeneously inside a transparent silicone flat sheet elastomer matrix, which acts as a host for the active OSL particles and allows optical access to the embedded OSL grains. Then, 2D OSL dose mapping is obtained using the self-developed optical detection setup consisting of a CCD camera and LEDs illuminating the prototype LMP-based foils. Next, the 3D dose distribution is obtained by building up stacks of 2D OSL images, providing fast and direct 3D spatial dose distribution measurement. Technology was tested to verify a real, clinical proton treatment plan prepared for eyeball cancer [12]. Although the obtained results showed good agreement with dose distributions calculated in a clinical treatment planning system, the LMP OSL material response to protons showed a very well-known effect of the so-called quenching or lower luminescent efficiency. This is similar to what has been observed for other types of OSL materials and was previously investigated, e.g., for AL_2_O_3_ [21], BeO [22], and CaF_2_ [23], and is related to the ionisation density of the protons’ particles that pass through the OSL material. The efficiency parameter depends on a given material energy response to protons and on radiation quality parameters, for instance, a spectrum of kinetic energy or linear energy transfer (LET) of protons [24,25]. Detailed knowledge of detector efficiency is crucial in establishing a calibration method for detectors exposed to mixed radiation fields produced by proton beams (or any other heavy ions) to correctly calculate the dose distribution and dosimetry verification procedure. It should be stated that even monoenergetic proton beams undergo scattering and straggling when interacting with matter, leading to a broadening of a kinetic energy spectrum over the volume of interest. Thus, proton fields, composed of beams with various energies (as it takes place for the spread-out Bragg peak, SOBP), have a wide energy spectrum; thus, detector efficiency may vary significantly. Therefore, it is crucial to validate experimentally not only a single Bragg curve produced by a monoenergetic proton beam but also the whole SOBP, where a mixture of kinetic energies creates a mixed radiation field.

Detector efficiency may be related to beam quality factors by performing various measurements under various, well-defined irradiation conditions. Therefore, the objective of the present study was to investigate the LMP OSL foil detector’s relative efficiency to protons, using both real proton irradiations and Monte Carlo simulations. To establish the LMP OSL material and beam quality factors leading to detector efficiency response, a comprehensive study of particle transport for proton beams and LMP-silicone-based samples, using numerical tools such as Monte-Carlo (MC) particle transport codes, has been prepared. These simulations correspond to a measurement campaign in which a stack of 40 LMP foils, placed in a specially designed PMMA phantom, was irradiated, first within monoenergetic 58.8 MeV proton Bragg peak (BP) conditions and later with a passively modulated spread-out Bragg peak (SOBP) of the same initial energy, where a mixture of energies created a mixed radiation field. The geometry setup of the irradiated LMP foils was implemented in MC code, including LMP foil detector positions and relevant passive beam modifiers, all to best mimic the conditions during the real proton irradiations. Several beam quality parameters were scored for such implemented simulation codes, including proton kinetic energy spectra. Finally, the correction factor of the relative efficiency parameter has been derived and applied to the LMP foils’ proton dose–response.

## 2. Materials and Methods

### 2.1. LMP-Based Foils and Optical Detection System

**Figure 1:**
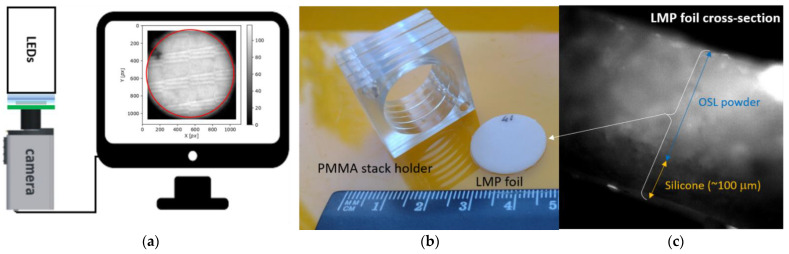
(**a**) Sketch of the optical setup for retrieving the 2D OSL dose distribution, consisting of the LED’s system, filters set together with the LMP foil, and the CCD camera. In the red circle, the captured 2D OSL image from the LMP sample foils has been shown. (**b**) The prototypes of the 2D OSL silicone foil of a size (20 ± 0.1) mm in diameter and (0.54 ± 0.3) mm thickness, together with the stack of PMMA holders with a hole the size of the LMP foil, used for proton irradiation. (**c**) Cross-section of the LMP foil illustrating the OSL powder distribution inside the silicone matrix.

The reported study was performed with the LMP-based, OSL silicone foils of a size of approximately (20 ± 0.1) mm in diameter and (0.54 ± 0.03) mm thickness, made by mixing the LMP powder grains (of a size of approx. 60 µm) homogeneously into a transparent silicone matrix, being the SYLGARD^®^ 184 Silicone Elastomer Kit from Dow Corning [26]. The silicone material is just a host for optically active OSL grains and allows optical access to the grains. Figure 1b shows an image of the silicone sample foils and a stack of the PMMA holders used for proton irradiations within this study. Figure 1c shows the OSL powder distribution in the silicone matrix. The observation was made using a Nikon Eclipse Ni–U upright fluorescence wide-field microscope with a DS-Qi2 CCD camera and a 20 × TU Plan ELWD objective lens. As shown in this figure, the OSL grains are distributed in the lower part of the detector (approx. 0.4 mm), while above them, a thin layer of approx. 0.1 mm silicone is visible. This is a result of gravitational descent before the congealing process ends. This finding has important implications for at least two issues. First, it should be considered when calculating the foil positions during the protons’ irradiations, especially with lower energies at the end of the BP and in the area of the distal fall-off, where protons stop in the detector volume. Second, image acquisitions should always be made in the same manner, i.e., the camera images face the same side of the detector.

The 2D OSL image acquisitions were performed using a self-developed optical detection set-up consisting of a high-sensitive CCD camera, a filter set for light discrimination, and 470 nm blue LEDs, which enlighten the foil area homogeneously during the acquisition time of 30 s (see Figure 1a). Images were acquired using the µManager open-source software accompanying the optical detection CCD camera setup [27]. To remove any pre-existing signal from each LMP foil after camera readout, a procedure was used comprising annealing at 200 °C for 60 min following stimulation with blue light for 24 h. A detailed description of the CCD camera optical detection setup and the LMP material synthesis and operation can be found in our previous study [9,10,11,12].

### 2.2. Method for Data Analysis of 2D OSL Images

Several improvements in the context of signal processing with respect to the method presented in our previous work [12] were implemented. For the clarity of the whole method, below we described all aspects, including the improvements reflected in the camera setup parameters, image distortions, and powder distribution inside the silicone matrix. Each of the captured images was corrected in the following procedure requiring a couple of datasets. The process included the following parts: images acquired during the irradiation of detectors with proton beams and respective background images and images acquired during irradiation of detectors with reference radiation (^60^Co) and respective background images needed to apply IRF and efficiency corrections. Live view images were acquired for all irradiations required to properly locate detector shapes in the 2D image white noise images needed to apply flat field corrections.

**Figure 2:**
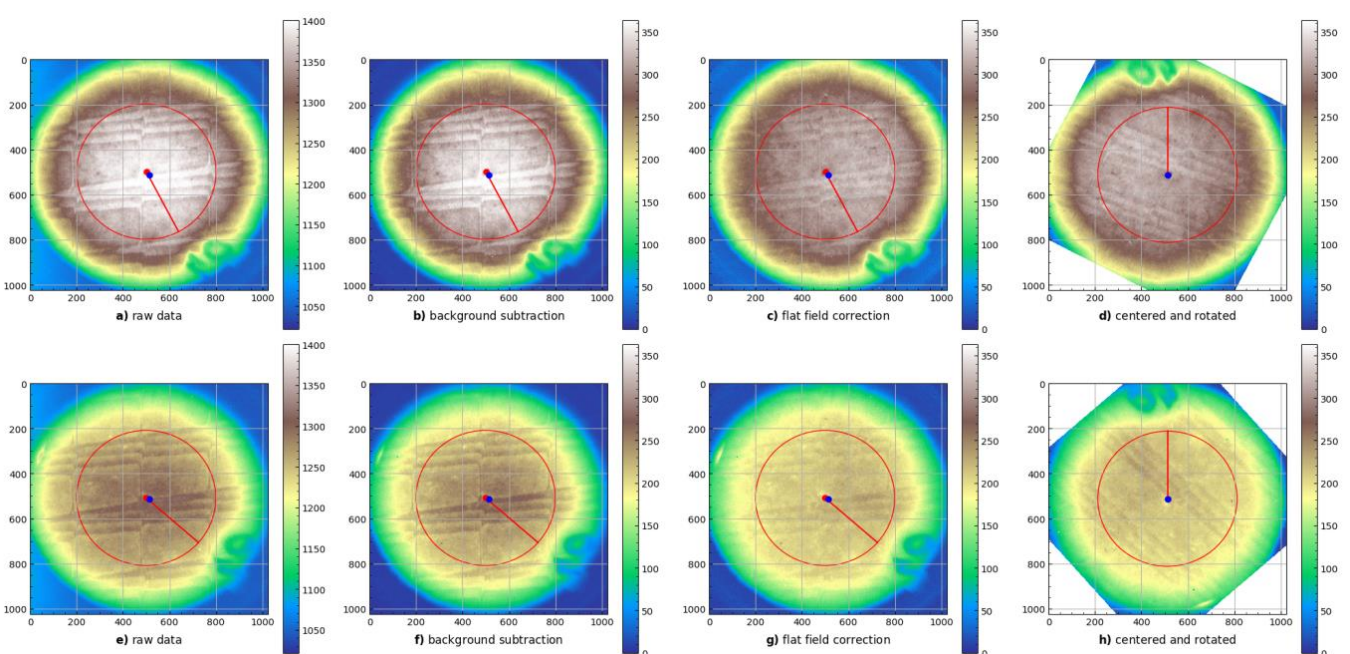
First steps for image correction procedure. Upper row (figures (**a**–**d**)) depicts detector irradiated in ^60^Co source, lower row (figures (**e**–**h**)) includes detector image irradiated with full modulation of proton beam (first detector); From left to right, the following processing steps are included: reading raw data, background subtraction, flat field correction, and detector alignment are included. The colour gradient denotes the pixel intensity, according to the colour scale presented to the right of each panel.

Data analysis was managed by Snakemake software (workflow management system [28]) and divided into several steps (Figure 2). After each processing step, intermediate data samples (images, numbers) are saved in the lossless NPY data format [29]. Below, there is a list of consecutive steps of the 2D OSL signal processing procedure:

1. Readout of raw data from the optical detection setup: Raw data are generated by the data acquisition software accompanying the CCD camera. The file format is a lossless TIFF file, with a resolution of 1024 × 1024, single channel, and 16 bits per sample. Improvement: No median filters, as we discovered that applying median filters introduces a bias into mean dose value.
2. Correction for increase in sensor stability with time: The readout system exhibits sensitivity increase at a rate of about 0.1 per minute. Improvement: raw data from signal and background images are adjusted by a correction factor deduced from the time difference between acquisition of the first and current image.
3. Background image subtraction from signal: Pixel-by-pixel subtraction of background image from the signal 2D image. Improvement: subtraction of per-detector background, instead of single background, for full dataset has been used.
4. Automatic discovery of detector location on live view images: The procedure is described in detail in [12]. We adjusted the threshold for contouring, switching from 30% of max value to 30% quantile. This works better for images containing hot spots.
5. Flat field correction: The aim of this step is to correct the artefacts introduced by the CCD sensor. A flat field image is acquired under the following procedure: first, an image with normal white light is acquired in 0.3 s without a real detector. Then, the gain factor is calculated as the flat field image normalised to the mean value in the central circle with radius of 300 px. Finally, the detector signal (after background removal) is multiplied by the gain factor.
6. Spatial alignment: The LMP detectors are placed at random angles and are not aligned in the readout system. Alignment is needed to obtain conditions necessary to combine data from ^60^Co and proton irradiations. The image is shifted in such a way that the detector is centred on the image: detector centre is located in the pixel with coordinates (512,512). Finally, the image is rotated by the angle necessary to move the marker to the top part of the image.

### 2.3. Monte-Carlo Simulation of Particle Transport

Monte Carlo (MC) simulations were performed with the PHITS Monte Carlo code (ver. 3.26) [30]. The following parts were included within the simulation procedure.

#### 2.3.1. Beam Source and Physics Models

For each setup, 10^6^ protons were simulated. The proton beam of the mean energy 58.4 MeV with FWHM = 1.4 MeV was simulated following the beam model described in [31]. An additional cut-off for the maximum proton kinetic energy equal to 58.55 MeV was applied. A cylindrical, parallel beam was used with a radius equal to 2 cm. The physics setup includes the Landau–Vavilov energy straggling for charged particles and nuclei, the Coulomb diffusion model by Lynch’s formula based on Moliere theory, and modelling of the transport of the photons, electrons, and positrons based on the EGS5 [32] algorithm. Additionally, the density correction based on ICRU90 [33] is performed. The energy cut-offs for protons (1 keV), electrons (0.1 MeV), positrons (100 keV), and photons (1 keV) were set.

#### 2.3.2. Simulation Geometry

**Figure 3:**
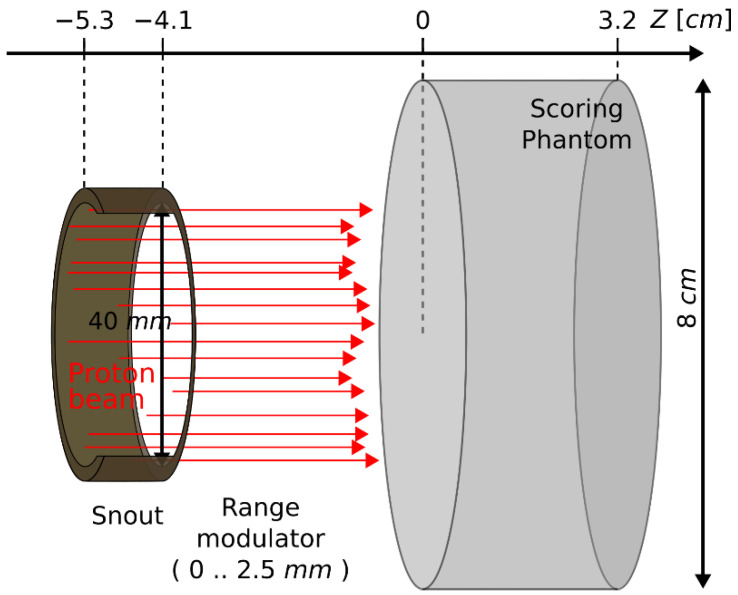
Simplified scheme of the Monte Carlo simulation geometry according to the description in Section 2.3.

Simplified geometry with cylindrical symmetry was used to assess depth–dose distribution and kinetic energy spectrum at several depths (Figure 3). Instead of detector material and PMMA plates, a cylinder of liquid water at a density of 1.0 g/cm^3^ was used. To achieve electronic equilibrium, the cylinder dimension was set to 8 cm. The depth of the cylinder was set to 3.2 cm, which is about 10% larger than the range of protons at 58.8 MeV.

In the case of SOBP geometry, a simplified representation of the modulator wheel was used. Slabs of PMMA (density 1.174 g/cm^3^) with varying thicknesses were placed between the beam source and the water phantom cylinder. For SOBP, appropriate weighting factors were used to combine several single-energy simulations. These weighting factors account for the decrease in proton fluence due to multiple Coulomb scatterings placed on the modulator wheel.

#### 2.3.3. Definition of Simulation Output (Scoring)

The dose profiles and kinetic energy of the protons (energy spectrum) were scored along the beam direction. To mimic the experiment, the same positions of the scoring in the detector were chosen. The cylindrical scoring was used to decrease the computational time with the cylinders, which all have 0.05 mm thickness. Moreover, a second type of dose scoring was performed. For the energy spectrum, information from all protons was stored in 600 equally distributed bins from 0–60 MeV for each of the experimental positions.

### 2.4. Relative Luminescence Efficiency—Definition

The relative luminescence efficiency (η) [1] is defined as the ratio of the emitted light intensity per unit dose for a given radiation type *k*, e.g., protons, to the same quantity for the reference radiation type *g* (^60^Co—gamma radiation) as follows:

(1) $$\eta =\frac{\eta_{k}}{\eta_{\gamma }}=\frac{OSL_{k}}{D_{k}}\cdot \frac{D_{\gamma }}{OSL_{\gamma }}$$

Here, D_k_ and D_γ_ denote doses for a given radiation type and for reference radiation, respectively. OSL_k_ and OSL_γ_ are light intensities of the OSL signal measured at the doses D_k_ and D_γ_, respectively [1,24,25]. The reference irradiation performed with a uniform gamma radiation source of ^60^Co was used, available from the Theratron 780 therapy machine [34] with a homogenous dose of 60 Gy.

The relative OSL efficiency depends on OSL material energy response to protons but as well as on radiation quality parameters, i.e., kinetic energy or linear energy transfer (LET) of protons. These quantities are calculated as averaged values when considering mixed-field irradiation; i.e., we consider averaged LET or averaged kinetic energy. The determination of the quantities appearing in Equation (1) is not straightforward. The efficiency parameter usually depends on the ionisation density. This results from high local doses in a given material exposed to densely ionising radiation and non-linearity of OSL characteristic responses at such high doses. This characteristic is commonly sub-linear, caused by simple saturation of available trapping or recombination centres in the irradiated material, which is needed for OSL phenomena to occur. For this reason, the relative OSL efficiency typically decreases with increasing ionisation density and is lower than unity (see Section 3.2). Especially in particle radiotherapy with complex fields, to cover the whole tumour size, the dose is deposited by particles with a wide range of energies within a spread-out Bragg peak (SOBP). In such cases, the luminescence efficiency depends on the actual protons’ energy spectrum reaching the detectors [2,24,25].

### 2.5. Proton Beam Dosimetry vs. Monte Carlo Code Implementation

**Figure 4:**
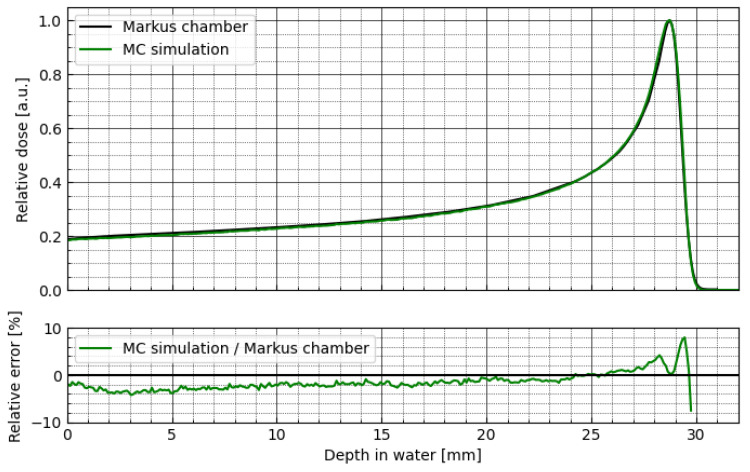
Comparison of the proton depth–dose distribution (the so-called pristine Bragg peak) obtained for the Markus ionisation chamber (solid black line) and calculated from the MC simulation (solid green line) for the proton energy used during the experiment with LMP foils (58.8 MeV), using the described above numerical MC code. The measured ionisation chamber parameters comprised range, R_90%_ = 28.96 mm, and distal fall-off Δ(R_90%_ − R_10%_) = 0.78 mm being the difference in the ranges of 90% and 10% of the Bragg peak. Symbol R indicates the range of protons in water (90% at the distal edge).

The LMP foils have been irradiated at the Proton Eye Radiotherapy Facility (Institute of Nuclear Physics—IFJ PAN) with a 58.8 MeV proton beam available from the AIC-144 isochronous cyclotron [34]. Proton beam dosimetry was carried out in a water phantom according to the IAEA TRS-398 protocol [35], by a plane-parallel Markus ionisation chamber model TM23343 connected to the reference class electrometer PTW UNIDOS [36]. Figure 4 compares the relative proton depth–dose distribution (the so-called pristine Bragg peak) measured by the Markus ionisation chamber (solid black line) in water for a 58.8 MeV BP used during the experiment with the LMP foils setup, compared with the dose distribution obtained from the MC simulation described in Section 2.3 (solid green line). The measured ionisation chamber parameters comprised proton range, with R_90%_ = 28.96 mm being the range of protons in water (90% at the distal edge), and distal fall-off Δ(R_90%_ − R_10%_) = 0.78 mm being the difference in the ranges of 90% and 10% of the Bragg peak. Symbol R indicates the proton energy after the passive scattering, corresponding to the proton range of 29 mm in water, and was calculated as 58.8 MeV. As the figure shows, within the BP range (up to 29 mm), the differences are less than 5%.

### 2.6. Experimental Phantom Used for the LMP Foils Proton Irradiation

**Figure 5:**
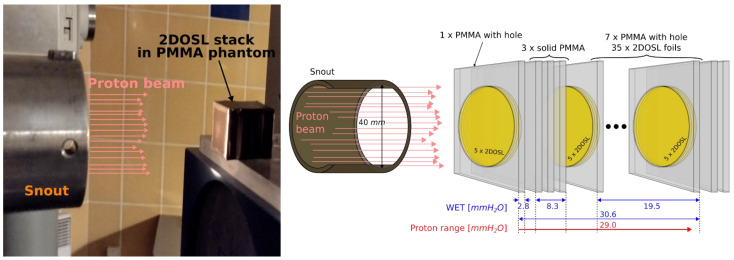
Experimental setup mounted on top of the therapeutic chair in the treatment room of the Proton Eye Radiotherapy Facility (IFJ PAN). The construction of the phantom comprised 40 silicone LMP foils attached to 13 PMMA holders with and without holes, irradiated with a 58.8 MeV proton beam available from the AIC-144 isochronous cyclotron [34].

Figure 5 shows the experimental setup used for all (PB and SOBP) proton irradiations within this study. A stack of 40 LMP foils has been placed inside a specially designed phantom made of PMMA holders with and without holes with similar diameter as the LMP foil (see also Figure 1b) so that the foils were irradiated at various depths in the whole BP and SOBP range. All proton irradiations were performed at room temperature. Considering the LMP foils thickness, 40 LMP samples do not cover the 58.8 MeV protons’ range, which is approx. 29 mm in the water range (see Figure 4). Thus, the foils were distributed within the PMMA phantom as follows: 5 LMP foils were stacked together and placed in one PMMA holder with a hole at the beginning; next, 3 PMMA solid plates were stacked, behind which the rest of the 35 LMP foils were stacked together using the PMMA holders with holes. The total thickness of such a constructed experimental phantom comprised 40 times 0.54 mm (average LMP foils thickness) and 7.2 mm thickness of the three PMMA plates. Taking into account the experimentally calculated water-equivalent thickness, the so-called WET parameter, being the thickness of the water layer, expressed in g/cm^2^, which causes the same loss of proton energy as in a given material with a given thickness, for the LMP foil material (WET = 1.05) and for the PMMA plate (WET = 1.15), the total available range for proton measurements of the experimental phantom expressed in water millimetres is fixed to 30.6 mm (see Figure 5).

## 3. Results and Discussion

### 3.1. Relative Efficiency Calculation Based on Irradiation with Monoenergetic Beam (BP)

Since the detector luminescence efficiency depends on radiation quality parameters (i.e., kinetic energy, in case of irradiation with protons), the detailed knowledge of detector efficiency is crucial in establishing a calibration method for LMP OSL detectors, especially exposed to mixed fields of protons, as it is taking place for SOBP proton irradiations [24].

**Figure 6:**
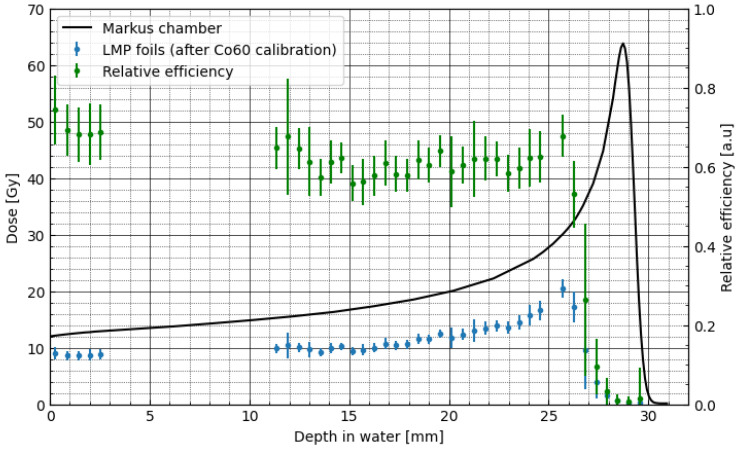
Comparison of the proton depth–dose distributions in water, measured with the Markus ionisation chamber (solid black line and left-hand scale) and with the LMP foils (blue points). LMP foils dose was calculated with applying ^60^Co calibration on signal from proton irradiation. The relative luminescence response, shown as green points (right-hand scale), was calculated as the ratio of the dose obtained from the Markus ionisation chamber and the LMP foils (according to Equation (1)). The dots represent the quantity calculated in the central circle of radius 300 pixels. Respectively, the error bars represent standard deviation. Data from a detector placed at 26 mm were rejected as outliers.

To derive the relative luminescence efficiency corrections, first, the experiment with pristine BP with the initial energy of 58.8 MeV and entrance dose of 12 Gy has been prepared. Figure 6 (left-hand scale) shows the comparison of the proton depth–dose distribution obtained for the Markus ionisation chamber (solid black line) and measured directly from the 40 LMP foils (blue points) placed at various depths of the BP, using the experimental setup described in Section 2.6. The obtained signal from the LMP foils (blue points), calculated with applying ^60^Co calibration on signal from proton irradiation, shows an under-response comparing the signal from the ionisation chamber. Figure 6 (green points and right-hand scale) shows the derived values, being the so-called luminescence efficiency (see Equation (1) in Section 2.4 and Appendix A), as the ratio of the uncorrected dose–response obtained from the captured 40 OSL signals of the LMP foils and directly from the Markus ionisation chamber.

### 3.2. The Relative Efficiency vs. Proton Energy Distribution Obtained from the MC Simulation

**Figure 7:**
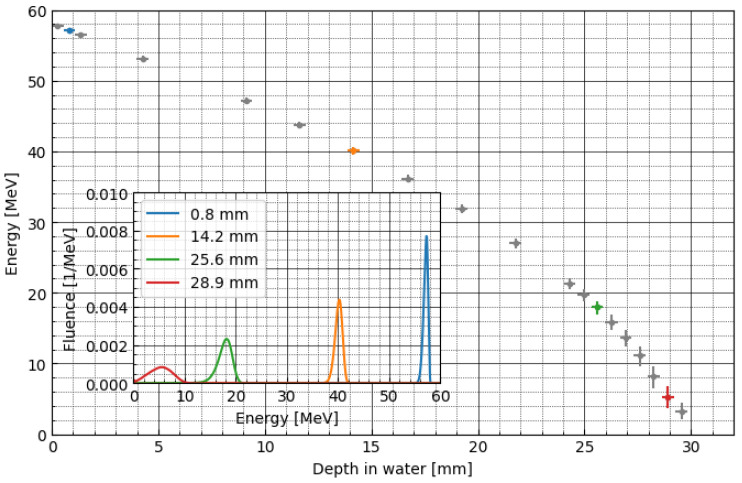
Depth profile of proton kinetic energy for monoenergetic proton beam. Circles represent the median of proton kinetic energy calculated in a slab representing LMP foil. The error bars represent interquartile range. The inset presents the energy spectra for selected depths.

**Figure 8:**
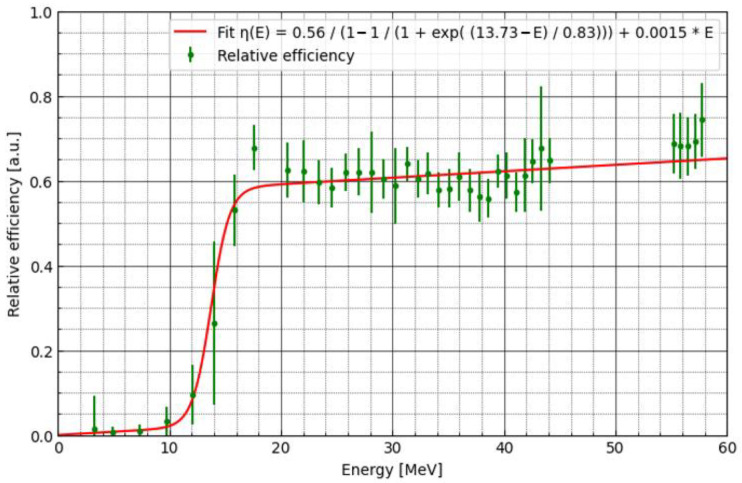
Comparison of the relative efficiency (green points)—depth–dose correction factor calculated according to Equation (1). Sigmoidal fit (composition of logistic and linear models) is presented as a red line.

To build the efficiency corrections, we need to apply an MC simulation, as the energy at a given point of the LMP foils’ position during proton irradiation cannot be measured directly. Figure 7 shows the proton energy spectra calculated using the PHITS particle transport code for the monoenergetic beam. Additionally, the inset graph shows the energy spectrum for selected depths. For the construction of the analytical model (fit) of correction factor, median kinetic energy was used, and interquartile range (IQR) was applied as weighting factor. Figure 8 shows the sigmoidal fit-to-depth correction factor calculated as the ratio of the relative dose measured by the Markus chamber and the LMP foils (blue points in Figure 6). The dose under-response presented in Figure 6 was corrected using the analytical model of the efficiency correction (presented on Figure 8, see also Appendix A)). The fitting model is a composite of the step and linear models (here, logistic function). The step model was chosen to represent a drop of efficiency as a certain energy threshold. The linear model was selected to consider the weak dependency of efficiency as a function of energy for values greater than 20 MeV. The exact step model was chosen after trying several options (error function, arcus tangens, logistic function) and selecting the one that has the best-fit quality parameters. The best fitted parameters can be used to describe the physical attributes of the efficiency model. The centre at 13.73 MeV gives the threshold level for the efficiency drop. The amplitude of the step model (0.56) is a main part of the efficiency at the highest energy being used. The slope of the linear model (0.0015) is related to weak dependency of the efficiency on energy in regions above 20 MeV, yielding fit quality chi^2^ = 13.74 (and reduced chi^2^ 0.4).

**Figure 9:**
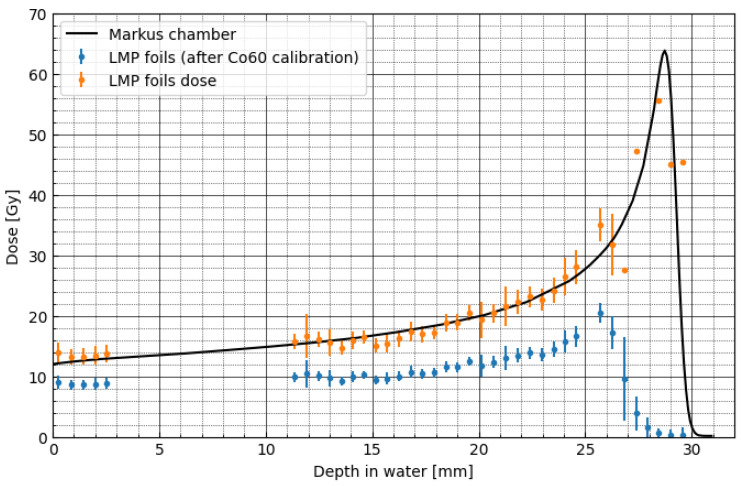
Comparison of the depth–dose profiles, measured for the Markus ionisation chamber (solid black line) and for the LMP foils obtained before (blue points) and after applying correction factors (orange points) for the monoenergetic proton beam. Points denote mean values; error bars represent standard deviation calculated on a circle with radius 300 px.

The corrected dose measured with LMP foils shows agreement under 10% with measurement conducted with the Markus chamber, as seen in Figure 9. Largest deviations are present at lowest proton energy (<14 MeV), corresponding to the 27 mm depth in water.

### 3.3. Luminescence Efficiency Correction for the Spread out Bragg Peak (SOBP)

**Figure 10:**
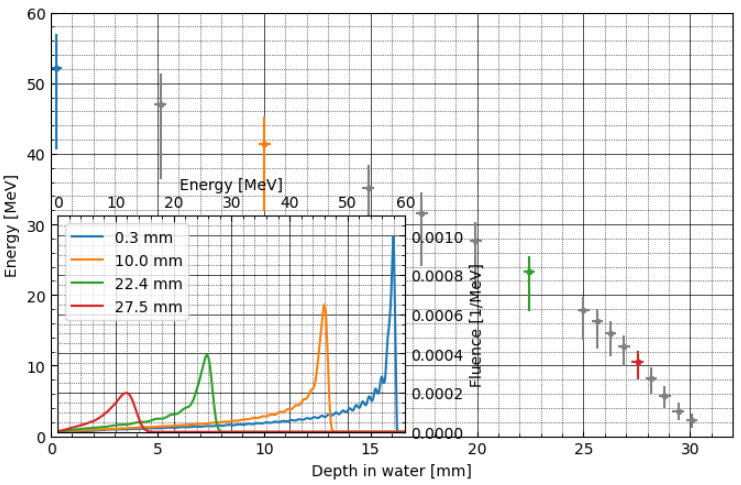
Depth profile of proton kinetic energy for spread-out proton beam (full modulation). Circles represent the median of proton kinetic energy calculated in a slab representing LMP foil. The error bars represent the interquartile range. The inset presents the energy spectra for selected depths.

Figure 10 shows the depth profile of proton kinetic energy and energy spectra for the spread-out Bragg peak (SOBP). It is observed that the median values for corresponding depths are lower than for monoenergetic proton beams. This is due to the contribution of multiple lower energetic beams with lower ranges of SOBP. The same contribution manifests itself in broadening of the energy spectra and a much larger tail at lowest energies.

**Figure 11:**
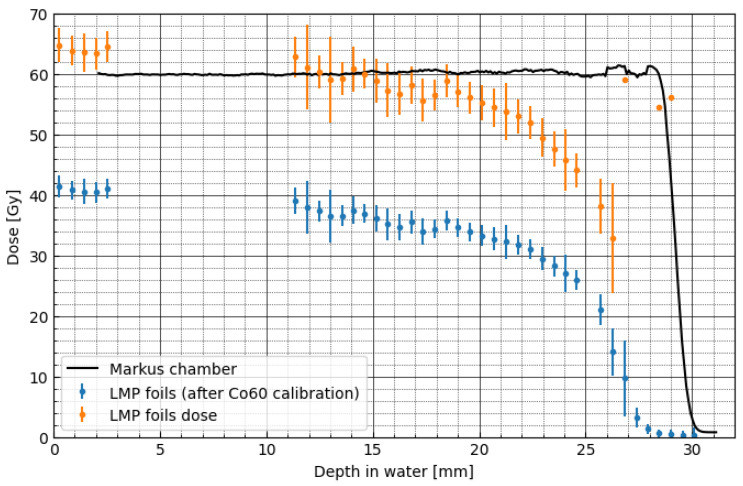
Comparison of the depth–dose profiles, measured for the Markus ionisation chamber (solid black line) and for the LMP foils obtained before (blue points) and after applying correction factors (orange points) for the spread-out proton beam. Points denote mean values; error bars represent standard deviation calculated on a circle with a radius 300 px.

We employ a similar procedure as described in Section 3.2 for pristine BP (seen in Figure 6) to correct dose under-response in spread-out proton beam (SOBP). The median kinetic energy and IQR calculated for SOBP (seen in Figure 10) and analytical efficiency correction model (seen in Figure 8) are employed to adjust the dose measured with LMP foils. The results are compared in Figure 11, as the depth–dose profiles, measured for the Markus ionisation chamber (solid black line) and for the LMP foils obtained before (blue points) and after applying correction factors (orange points). As a consequence, we observe 10% agreement between LMP dose and Markus chamber measurements for depths up to 21 mm (corresponding to kinetic energy of 26 MeV). For deeper regions, the LMP dose is significantly lower than ionisation chamber readouts. This phenomenon cannot be explained with the currently presented approach and poses an interesting problem for further studies. A simple explanation can be made. The energy spectra of the monoenergetic beam have a symmetrical distribution for which the maximum value matches the median kinetic energy, which is not the case for spread-out beams where contributions from lower energetic sub-peaks create a significant tail. Such a spectrum is highly skewed, and the median value does not match the central point of the highest peak. The impact of the spectrum on the derived corrections and relative efficiency is more complex. As we learn from experiments with monoenergetic beams, there is a very weak dependency of relative efficiency on energy for regions above 20 MeV. This region corresponds to depths in water lower than about 25 mm. In that region, we assume that the energy spectrum shape also has minimum impact on relative efficiency. On the other hand, the situation is different for lower energies (below 20 MeV, residual range about 4.2 mm). We expect lower efficiency for the SOBP than in monoenergetic beams due to the skewness of the energy spectrum, which tends towards lower energies. The effect is not straightforward as, due to the relatively large thickness of the detector (WET = 0.55 mm), most of the lower energy protons either stop or lose a significant part of kinetic energy in the detector volume. A more sophisticated approach or thinner detector could improve the correction applied to the spread-out depth–dose profile.

## 4. Conclusions

In the present work, a comprehensive research effort dedicated to the characterization of the OSL relative response of the LiMgPO_4_ (LMP) silicone foils to protons and its dependence on proton energy has been presented. In the experimental part of the study, the proton depth–dose distribution was measured for the pristine monoenergetic BP and modulated SOBP. We employed 40 LMP flat sheet silicone foils stacked together inside a specially designed PMMA phantom. The irradiation took place in the therapeutic room of the proton eye radiotherapy facility. The ^60^Co gamma source in the same facility was used as the reference radiation. To derive response of the LMP OSL material to the proton beams, we employed an approach based on beam quality factors calculated using particle transport codes. The simulated proton beam was characterised by the depth profile of the dose and kinetic energy spectra. The obtained data confirmed the occurrence of the LMP under-response to protons (also known as the quenching effect), being related to the ionisation density of the protons that pass through the LMP material. Although the signal for the monoenergetic BP was approx. 40% lower than the dose measured with the Markus chamber, the applied LMP dose–response correction procedure minimises the influence of the under-response. A sigmoidal model of the efficiency correction factor was obtained from experimental and simulated data. We observed a significant drop of the efficiency at energies of about 13.4 MeV (corresponding to a residual range of 2.07 mm) from the level of 0.65 down to values below 0.05. The efficiency correction factors have also been applied for the complex SOBP proton beam. The obtained results improve LMP dose–response at depths up to 21 mm (within the 10% agreement level with dosimetric data). This improvement trend is still present for the larger depths, however less pronounced. Thoroughly explaining the obtained data with the currently presented approach is not straightforward. However, this opens an interesting problem for further studies.

## Data Availability

The computer code used in data analysis and interim data files are freely available in the public repository https://github.com/grzanka/ifj_lmp_foils (accessed on 23 February 2023). Raw data are available upon request.

## Appendix A

Example of the methodology of the relative efficiency calculation: The relative efficiency of the OSL material has been investigated in the experiment with a monoenergetic proton beam that creates a single Bragg depth–dose curve and is shown in Figure 6. We calculated the relative efficiency according to the definition in Equation (1). The efficiency was then presented as a function of the median proton kinetic energy at a given depth, calculated with the Monte Carlo particle transport code. We used such calculated relative efficiency to correct the response of 2D OSL foils for SOBP irradiation, where the median kinetic energy was also calculated. As an example, let us consider a detector placed in the entrance region of the depth–dose curve. The calibration procedure was performed under D_γ_ = 60 Gy and yielded OSL_γ_ = 306.5. During the irradiation with protons, the same detector was exposed to dose D_k_ = 12 Gy and yielded OSL_k_ = 46. Putting those numbers in Equation (1), we obtain relative efficiency 0.75. The same procedure was applied to all detectors in stack and allowed us to derive an analytical formula for the relative efficiency, as shown in Figure 8:

*η*(E) = 0.56/(1 − 1/(1 + exp(13.73 − E)/0.83)) + 0.0015 * E
(A1)

To apply this correction on the irradiation in the mixed field (so called SOBP), we used Monte Carlo particle transport code to derive median kinetic energy in the position of the detector. This gives us 57.75 MeV. Applying the analytical formula (A1), we obtain η(E) = 0.65.

Using again Equation (1), we can derive dose for protons D_k_ as:

D_k_ = OSL_k_/*η* * D_γ_/OSL_γ_
(A2)

During SOBP irradiation, the detector emitted light with intensity OSL_k_ = 211.4. Median kinetic energy was equal to 52.15 MeV. Finally, we can apply efficiency correction as follows:

D_k_ = OSL_k_/*η*(52.15) * D_γ_/OSL_γ_ = 64.7 Gy
(A3)

## References

[1] Yukihara E.G., McKeever S.W.S.M. (2011). Optically Stimulated Luminescence: Fundamentals and Applications.

[2] Yukihara E.G., Bos A.J.J., Bilski P., McKeever S.W.S. (2022). The quest for new thermoluminescence and optically stimulated luminescence materials: Needs, strategies and pitfalls. Radiat. Meas..

[3] Gasparian P.B.G., Malthez A.L.M.C., Campos L.L. (2022). Using the optically stimulated luminescence technique for one- and two-dimensional dose mapping: A brief review. Phys. Med. Biol..

[4] Idri K., Santoro L., Charpiot E., Herault J., Costa A., Ailleres N., Delard R., Vaille J., Fesquet J., Dusseau L. (2004). Quality control of intensity modulated radiation therapy with optically stimulated luminescent films. IEEE Trans. Nucl. Sci..

[5] Jahn A., Sommer M., Liebmann M., Henniger J. (2011). Progress in 2D-OSL-dosimetry with beryllium oxide. Radiat. Meas..

[6] Li H.H., Driewer J.P., Han Z., Low D.A., Yang D., Xiao Z. (2014). Two-dimensional high spatial-resolution dosimeter using europium doped potassium chloride: A feasibility study. Phys. Med. Biol..

[7] Nyemann J.S., Turtos R.M., Julsgaard B., Muren L.P., Balling P. (2020). Optical characterization of LiF:Mg,Cu,P—Towards 3D optically stimulated luminescence dosimetry. Radiat. Meas..

[8] Ahmed M.F., Shrestha N., Ahmad S., Schnell E., Akselrod M.S., Yukihara E.G. (2017). Demonstration of 2D dosimetry using Al_2_O_3_ optically stimulated luminescence films for therapeutic megavoltage X-ray and ion beams. Radiat. Meas..

[9] Sądel M., Bilski P., Sankowska M., Gajewski J., Swakoń J., Horwacik T., Nowak T., Kłosowski M. (2020). Two-dimensional radiation dosimetry based on LiMgPO_4_ powder embedded into silicone elastomer matrix. Radiat. Meas..

[10] Sadel M., Høye E.M., Skyt P.S., Muren L.P., Petersen J.B.B., Balling P. (2017). Three-dimensional radiation dosimetry based on optically-stimulated luminescence. J. Phys. Conf. Ser..

[11] Sądel M., Bilski P., Kłosowski M., Sankowska M. (2020). A new approach to the 2D radiation dosimetry based on optically stimulated luminescence of LiF:Mg,Cu,P. Radiat. Meas..

[12] Sądel M., Gajewski J., Sowa U., Swakoń J., Kajdrowicz T., Bilski P., Kłosowski M., Pędracka A., Horwacik T. (2021). 3d dosimetry based on limgpo4 osl silicone foils: Facilitating the verification of eye-ball cancer proton radiotherapy. Sensors.

[13] Dhabekar B., Menon S.N., Alagu Raja E., Bakshi A.K., Singh A.K., Chougaonkar M.P., Mayya Y.S. (2011). LiMgPO_4_:Tb,B—A new sensitive OSL phosphor for dosimetry. Nucl. Instrum. Methods Phys. Res. Sect. B Beam Interact. Mater. At..

[14] Menon S.N., Dhabekar B., Alagu Raja E., Chougaonkar M.P. (2012). Preparation and TSL studies in Tb activated LiMgPO_4_ phosphor. Radiat. Meas..

[15] Kumar M., Dhabekar B., Menon S.N., Chougaonkar M.P., Mayya Y.S. (2011). LiMgPO_4_:Tb,B OSL phosphor—CW and LM OSL studies. Nucl. Instrum. Methods Phys. Res. Sect. B Beam Interact. Mater. At..

[16] Kumar M., Dhabekar B., Menon S.N., Bakshi A.K., Udhayakumar J., Chougaonkar M.P., Mayya Y.S. (2013). Beta response of LiMgPO_4_:Tb,B based OSL discs for personnel monitoring applications. Radiat. Prot. Dosim..

[17] Kellerman D.G., Medvedeva N.I., Kalinkin M.O., Syurdo A.I., Zubkov V.G. (2018). Theoretical and experimental evidences of defects in LiMgPO_4_. J. Alloys Compd..

[18] Marczewska B., Bilski P., Wróbel D., Kłosowski M. (2016). Investigations of OSL properties of LiMgPO_4_:Tb,B based dosimeters. Radiat. Meas..

[19] Marczewska B., Sas-Bieniarz A., Bilski P., Gieszczyk W., Kłosowski M., Sądel M. (2019). OSL and RL of LiMgPO_4_ crystals doped with rare earth elements. Radiat. Meas..

[20] Gieszczyk W., Bilski P., Mrozik A., Kłosowski M., Marczewska B., Sas-Bieniarz A., Perzanowski M., Zorenko T., Zorenko Y. (2020). Intrinsic and Dopant-Related Luminescence of Undoped and Tb Plus Tm Double-Doped Lithium Magnesium Phosphate (LiMgPO_4_, LMP) Crystals. Materials.

[21] Yukihara E.G., McKeever S.W.S. (2006). Ionisation density dependence of the optically and thermally stimulated luminescence from Al_2_O_3_:C. Radiat. Prot. Dosim..

[22] Sommer M., Jahn A., Henniger J. (2008). Beryllium oxide as optically stimulated luminescence dosimeter. Radiat. Meas..

[23] Malthez A.L.M.C., Marczewska B., Ferreira F., Umisedo N.K., Nowak T., Bilski P., Yoshimura E.M. (2018). OSL dosimetric properties and efficiency of Brazilian natural calcium fluoride pellets. Appl. Radiat. Isot. Incl. Data Instrum. Methods Use Agric. Ind. Med..

[24] Yukihara E.G., McKeever S.W.S., Andersen C.E., Bos A.J.J., Bailiff I.K., Yoshimura E.M., Sawakuchi G.O., Bossin L., Christensen J.B. (2022). Luminescence dosimetry. Nat. Rev. Methods Prim..

[25] Dietze G., Bartlett D.T., Cool D.A., Cucinotta F.A., Jia X., McAulay I.R., Pelliccioni M., Petrov V., Reitz G., Sato T. (2013). ICRP Publication 123: Assessment of Radiation Exposure of Astronauts in Space. Ann. ICRP.

[26] Høye E.M., Sadel M., Kaplan L., Skyt P.S., Muren L.P., Petersen J.B.B., Swakoń J., Mierzwińska G., Rydygier M., Malinowski L. (2017). First 3D measurements of proton beams in a deformable silicone-based dosimeter. J. Phys. Conf. Ser..

[27] Schindelin J., Arganda-Carreras I., Frise E., Kaynig V., Longair M., Pietzsch T., Preibisch S., Rueden C., Saalfeld S., Schmid B. (2012). Fiji: An open-source platform for biological-image analysis. Nat. Methods.

[28] Mölder F., Jablonski K.P., Letcher B., Hall M.B., Tomkins-Tinch C.H., Sochat V., Forster J., Lee S., Twardziok S.O., Kanitz A. (2021). Sustainable data analysis with Snakemake. F1000Research.

[29] Numpy. https://numpy.org/devdocs/reference/generated/numpy.lib.format.html.

[30] Sato T., Iwamoto Y., Hashimoto S., Ogawa T., Furuta T., Abe S., Kai T., Tsai P.-E., Matsuda N., Iwase H. (2018). Features of Particle and Heavy Ion Transport code System (PHITS) version 3.02. J. Nucl. Sci. Technol..

[31] Parisi A., Olko P., Swakoń J., Horwacik T., Jabłoński H., Malinowski L., Nowak T., Struelens L., Vanhavere F. (2020). Modeling the radiation-induced cell death in a therapeutic proton beam using thermoluminescent detectors and radiation transport simulations. Phys. Med. Biol..

[32] Hirayama H., Namito Y., Bielajew A.F., Wilderman S.J., Nelson W.R. (2005). The EGS5 Code System. SLAC-R-730 (2005) and KEK Report 2005-8 Japan.

[33] ICRU (2016). Key data for Ionizing-Radiation Dosimetry: Measurement Standards and Applications. J. ICRU.

[34] Swakon J., Olko P., Adamczyk D., Cywicka-Jakiel T., Dabrowska J., Dulny B., Grzanka L., Horwacik T., Kajdrowicz T., Michalec B. (2010). Facility for proton radiotherapy of eye cancer at IFJ PAN in Krakow. Radiat. Meas..

[35] IAEA (2001). Absorbed Dose Determination in External Beam Radiotherapy an International Code of Practice for Dosimetry Based on Standards of Absorbed Dose to Water.

[36] PTW Dosimetry. https://www.ptwdosimetry.com/en/overview-pages/detectors-for-proton-dosimetry/.

